# Temperature and Oxidative Stress as Triggers for Virulence Gene Expression in Pathogenic *Leptospira* spp.

**DOI:** 10.3389/fmicb.2017.00783

**Published:** 2017-05-09

**Authors:** Tricia Fraser, Paul D. Brown

**Affiliations:** ^1^Department of Basic Medical Sciences, Biochemistry Section, University of the West IndiesMona, Jamaica; ^2^Veterinary Services Division, Ministry of AgricultureHope Gardens, Jamaica

**Keywords:** *Leptospira*, virulence, temperature, oxidative stress, regulation

## Abstract

Leptospirosis is a zooanthroponosis aetiologically caused by pathogenic bacteria belonging to the genus, *Leptospira*. Environmental signals such as increases in temperatures or oxidative stress can trigger response regulatory modes of virulence genes during infection. This study sought to determine the effect of temperature and oxidative stress on virulence associated genes in highly passaged *Leptospira borgpeterseneii* Jules and *L. interrogans* Portlandvere. Bacteria were grown in EMJH at 30°C, 37°C, or at 30°C before being transferred to 37°C. A total of 14 virulence-associated genes (*fliY, invA, lenA, ligB, lipL32, lipL36, lipL41, lipL45, loa22, lsa21, mce, ompL1, sph2*, and *tlyC*) were assessed using endpoint PCR. Transcriptional analyses of *lenA*, *lipL32*, *lipL41*, *loa22*, *sph2* were assessed by quantitative real-time RT-PCR at the temperature conditions. To assess oxidative stress, bacteria were exposed to H_2_O_2_ for 30 and 60 min with or without the temperature stress. All genes except *ligB* (for Portlandvere) and *ligB* and *mce* (for Jules) were detectable in the strains. Quantitatively, temperature stress resulted in significant changes in gene expression within species or between species. Temperature changes were more influential in gene expression for Jules, particularly at 30°C and upshift conditions; at 37°C, expression levels were higher for Portlandvere. However, compared to Jules, where temperature was influential in two of five genes, temperature was an essential element in four of five genes in Portlandvere exposed to oxidative stress. At both low and high oxidative stress levels, the interplay between genetic predisposition (larger genome size) and temperature was biased towards Portlandvere particularly at 30°C and upshift conditions. While it is clear that expression of many virulence genes in highly passaged strains of *Leptospira* are attenuated or lost, genetic predisposition, changes in growth temperature and/or oxidative intensity and/or duration were factors which acted in isolation or together with other regulatory cues to contribute to the variable gene expression observed in this study. Overall, differential gene expression in serovar Portlandvere was more responsive to temperature and oxidative stress.

## Introduction

Leptospirosis is a zooanthroponosis, widely distributed throughout the world and aetiologically caused by pathogenic bacteria belonging to the genus, *Leptospira* (Bharti et al., 2003; Pappas et al., 2008). Pathogenic *Leptospira* species are invasive and infection results from their ability to colonize and invade the renal tubes of incidental hosts. While the complete mechanism involved in leptospiral pathogenicity is not fully elucidated, several studies on leptospiral virulence and virulence-associated factors indicate the involvement of haemolysins, adhesins, heat shock proteins, flagellins/motility, lipopolysaccharide (LPS), catalase KatE, heat-inducible ClpB chaperone, and several outer membrane proteins (Artiushin et al., 2004; Cullen et al., 2005; Barbosa et al., 2006; Nally et al., 2007; Dong et al., 2008; Lourdault et al., 2011; Andrade and Brown, 2012; Eshghi et al., 2012).

Pathogenicity is multifactorial, requiring integrated mechanisms and pathways to establish an infection. As diverse pathogenic bacteria share common strategies to cause disease and infection, the extent of damage to host tissue is determined by multiple gene or gene products involved in pathways of signal transduction, invasiveness and toxigenesis. Bacteria rely on the ability to sense and respond to environmental cues, including changes in temperature, pH, osmolarity, oxygen availability, and nutrient conditions (Thomas and Wigneshweraraj, 2014). The changing environment, often influenced by climate change, prompts adaptation, and regulatory responses to enhance the survival of the pathogen, and by extension, the ability to cause infection is largely due to the pathogen’s ability and adaptability (Reis et al., 2008; Lau et al., 2010; Batchelor et al., 2012).

The state of physiological imbalance between the natural or exogenous production of and/or exposure to high levels of oxidants and the organism’s ability to counteract their harmful effects induce oxidative stress (Fulda et al., 2010). In biological systems, oxidation reactions involving organic molecules usually generate unstable free radicals including reactive oxygen species (ROS) and reactive nitrogen species (RNS) (Nathan and Shiloh, 2000; Seis, 2014). Unequivocal interplay between oxidant and anti-oxidant countermeasures trigger a myriad of cascades that are likely to induce oxidative stress. For example, in *Escherichia* coli, the presence of as little as 0.5 μM H_2_O_2_, regardless of the source, can be cytotoxic (Sobota and Imlay, 2011). Unlike enteric bacteria, saprophytic leptospires lack the two main transcriptional regulators of oxidative stress response in enteric bacteria, OxyR and SoxRS (Anjem and Imlay, 2012). However, *Leptospira interrogans* possesses four predicted FurR homologs and PerR, a negative peroxide regulator with sensitivity to low H_2_O_2_ levels (Fillat, 2014), which exhibits similarity to PerR in *Bacillus subtilis* which controls *katA* and *ahpC* expression (Lo et al., 2010). Other leptospiral defense mechanisms against oxidative stress involve but are not limited to peroxiredoxin LinAhpC, catalase KatE in *L. interrogans* compared to KatG in *L. biflexa*, heat inducible ClpB chaperone, and glutathione and thiol peroxidases, among others which function in the capacity as metalloproteins (Lourdault et al., 2011; Eshghi et al., 2012).

Adaptation by an organism during serial passage is well established since the generation of live attenuated vaccines (Gamberini et al., 2005). In most instances, gene expression among pathogenic bacteria, including *Leptospira* is attenuated in highly passaged cultures and associated with loss of or attenuated virulence. Cullen et al. (2002) noted concomitant changes in leptospiral surfaceome, colonial morphology and loss of virulence associated with highly passaged *Leptospira*. Other studies have reported observations of non-synonymous variant alleles (Lehmann et al., 2015), attenuation of genes involved in invasion (Toma et al., 2014) and plasminogen binding (Vieria et al., 2009). Gene function and virulence may be restored by passage through a host and/ or activation by stimuli.

Temperature and oxidative stress represent two of the main external (host and environmental) stresses which influence virulence and viability of pathogenic *Leptospira*. Given that *L. interrogans* serovar Portlandvere and *L. borgpetersenii* serovar Jules account for more than 60% of cases of human leptospirosis in Jamaica, and with the paucity of information regarding molecular pathogenicity and the drivers of virulence in these species, this study sought to determine the effect of temperature and oxidative stress on virulence associated genes in *L. borgpeterseneii* Jules and *L. interrogans* Portlandvere.

## Materials and Methods

### Bacterial Strains and Culture Conditions

*Leptospira interrogans* serovar Portlandvere strain MY1039 and *L. borgpetersenii* serovar Jules strain jules were sub-cultured biweekly into liquid Ellinghausen-McCullough-Johnson-Harris (EMJH) medium at 30°C (Cameron, 2015), to yield highly passaged cultures with >200 serial passages. Leptospires were visualized using an Olympus BX 53 darkfield microscope.

### Temperature and Oxidative Stress Conditions

Bacteria were grown in supplemented EMJH liquid medium to a density of 1 × 10^8^ bacteria per mL and subsequently pelleted via centrifugation at 3,200 × *g* for 15 min. The pelleted bacteria were washed three times with EMJH medium and the bacteria were then re-suspended in EMJH medium and visualized via darkfield microscopy. One milliliter of cultures (3 × 10^8^ cells) was used to seed each 50 mL aliquot of fresh growth medium which was incubated either at 30°C for 14 days, 37°C for 14 days, or 30°C for 7 days before transfer to 37°C for an additional 7 days to simulate upshifted temperature conditions. Biological replicates (rather than technical replicates) were used at each temperature condition. These sets of conditions established the baseline for further comparisons. Following incubation, bacteria were visualized via darkfield microscopy to ensure bacterial viability prior to exogenous oxidative stress. Hydrogen peroxide (H_2_O_2_) was added to inoculated media at a final concentration of 1 mM or 10 mM and samples were incubated for 30 or 60 min at the previously incubated temperatures (Eshghi et al., 2012). Four exposure conditions ensued: 1 mM H_2_O_2_ for 30 min; 10 mM H_2_O_2_ for 30 min; 1 mM H_2_O_2_ for 60 min; and 10 mM H_2_O_2_ for 60 min. Controls without H_2_O_2_ were also analyzed. Following exposures, cells were collected by centrifugation for subsequent analyses.

### DNA Isolation and Endpoint PCR Analysis of Virulence Associated Genes

Genomic DNA was isolated from resuspended pellets by using the DNeasy blood and tissue kit (Qiagen, CA, USA) as per the manufacturer’s instructions. DNA quality was assessed by electrophoresis and quantification done using the Qubit 3.0 fluorometer (Invitrogen, USA) and Qubit dsDNA BR Assay Kit (Invitrogen, USA). Each biological replicate was done in duplicate. Endpoint PCR was performed to confirm the presence of open reading frames for the 14 virulence-associated genes investigated, which included genes for outermembrane proteins (*lipL36, lipL41, lipL45*), genes involved in adherence (*lenA, ligB, lipL32, loa22, lsa21, ompL1*), invasion (*invA, mce*), haemolysis (*sph2, tlyC*), and motility/chemotaxis (*fliY*). Two nanogrammes of DNA were used for amplification in a total reaction volume of 25 μL containing final concentration of 25 mM MgCl_2_, 500 μM of each deoxynucleotide triphosphate (dNTP), 5 U Taq polymerase, and 5 μM of each primer listed in **Table 1**. Primers were synthesized by Integrated DNA Technologies (IDT, IA, USA). Each biological replicate was done in duplicate and amplifications were carried out in a Techne TechGene Peltier thermal cycler and products separated on ethidium bromide-stained agarose gels.

**Table 1:** Primer sequences and annealing temperatures used in endpoint PCR and RT-PCR in this study.

Primers	Sequence (5′→3′)	Annealing temperature (°C)
fliY-F	ATGGGTGAAGGTTCCCTATCACAG	
fliY-R	TCACTTACCCTCCGGCTTAATCCG	
		49
ligB-F	CAGATATTCTTACCGTTTCCAATACA	
ligB-R	ATATCCGGAATGAATTTTGGTGTAAA	

lipL41-F	ATGAGAAAATTATCTTCTCTA	
lipL41-R	TTACTTTGCGTTGCTTTCGTC	
		54
lipL36-F	TTAACGAGATCTAAAAGTGACGATGAT	
lipL36-R	CATGATAAAAATTGAAAATGATTCAAGAAT	

lenA-F	CTGGAGTATTCGTGTGGGGATAAA	
lenA-R	CCATGGTAGAAATCAAACATCGCC	
		56
loa22-F	TTGTTGTGGTGCGGAAGTCG	
loa22-R	GGTCCCGAACAAGCAGAAGG	

invA-F	GACAAACCCTACCGA	
invA-R	CGATCTATTTCCGATGTC	
lipL32-F	GTGCTTTCGGTGGTCTGC	
lipL32-R	TTACTTAGTCGCGTCAGA	
lipL45-F	AGTTCCAAGGCAGCCGCTACTA	
lipL45-R	ATCATATAGGCGGAATTTAG	
		58
mce-F	AATATGAATTCGTTA	
mce-R	AAAAGCACTTAAGGCAGC	
ompL1-F	ATCCGTAACAATAGTAAG	
ompL1-R	GAGTTCGTGTTTATAACC	
spH2-F	TTACCCGAAAAAGAATCCTC	
spH2-R	TCCGGATTTAAGAGGCCAGG	
tlyC-F	ACATCTTTTCTTTTGAAGCTGATTGG	
tlyC-R	ACATCTTTTCTTTTGAAGCTGATTGG	

lsa21-F	GATGAAAAAAAAGAAAATGAATTGAG	
		60
lsa21-R	CTTCGCAACTTGTGGATAAGG	


### RNA Isolation, Endpoint RT-PCR, and Quantitative RT-PCR Analyses

Total RNA was isolated from resuspended bacterial pellets using TRIzol LS reagent (Invitrogen) and RNA was purified according to the manufacturer’s instructions. Purified RNA was reconstituted in RNase-free water and any contaminating DNA was removed by treating with Turbo DNase (Ambion, TX, USA) following the manufacturer’s recommendations. RNA was quantified using Qubit 3.0 fluorometer (Invitrogen) and Qubit RNA BR Assay Kit (Invitrogen). cDNA synthesis (reverse transcription at 50°C for 30 min followed by inactivation at 95°C for 15 min) of RNA extracts was performed using the OneStep RT-PCR Kit (Qiagen) in a total volume of 25 μL, with 1 μg total RNA and 0.6 μM of each primer (listed in **Table 1**), and components of OneStep RT-PCR enzyme mix with Omniscript and Sensiscript reverse transcriptases, based on the manufacturer’s instructions. Endpoint RT-PCR was performed to confirm transcription of the genes being investigated and each biological replicate was done in duplicate.

Quantitative RT-PCR (qRT-PCR) analyses were conducted using custom Taqman Gene Expression assays with fluorescent reporter dye, 6-carboxy-fluorescein (FAM)-labeled primer pairs and probe and Taqman Fast Virus 1-step master mix (Applied Biosystems, CA, USA). Virulence-associated genes analyzed included *lenA, lipL32, lipL41, loa22, and sph2*. These were selected for further study based on their consistent expression in both species in the previous endpoint RT-PCR analyses. The probes were designed using the software programme Primer Express^TM^ (Applied Biosystems), for compatibility with primer sequences used in endpoint RT-PCR: *lipL32* (FAM-CCAGGGACAAACGAA-MGBNFQ), *lipL41* (FAM-ATCAGATGCCTTCTAAAG-MGBNFQ), *loa22* (FAM-CGCAGAAGCAAACA-MGBNGQ), *lenA* (FAM-AGTTTAACGGGAGCTTAT-MBGNFQ), and *sph2* (AM-CACGCTCAACCACC-MGBNFQ). Taqman primer pairs and probes were synthesized by Applied Biosystems in a custom gene expression assay. The labeled MGB probe had the FAM located at the 5′ end of the probe and a non-fluorescent quencher (NFQ) at the 3′ end. For qRT-PCR, cDNA was synthesized in a total reaction volume of 20 μL containing 0.1 μg total RNA with components of the 4x Taqman Fast Virus 1-Step Master mix (Applied Biosystems), 20x Taqman Custom gene expression assay and RT-PCR grade H_2_O, to provide a final concentration of 5 μM labeled probe and 18 μM of each primer. Amplification was done as singleplex reactions with each biological replicate being amplified in duplicate. Controls with each run included a no-template control (NTC) that contained all the listed reagents except the RNA template and a no-enzyme control to detect the presence of contaminating DNA. Thermal cycling was performed in a 7500 Fast Real-Time PCR System (Applied Biosystems), using the following parameters: reverse transcription at 50°C for 5 min, inactivation at 95°C for 20 s, followed by 40 cycles at 95°C for 3 s and 60°C for 30 s. Gene expression results were reviewed for run validity. Negative reactions were assigned where no amplification occurred at threshold cycle (C_T_) greater than 38 cycles. The gene expression data (gene abundance) from the qPCR experiments were means of duplicate biological replicates quantified based on a 3-point standard curve; these were relative values of pathogenic leptospiral total RNA. Because of the dispersion of the data, it was necessary to transform them using logarithm base-10. Analysis of Variance (ANOVA) was used to evaluate the differential expression. Expression data for the five genes were normalized using gene expression values the 16S rRNA gene at 30°C for the two strains and analyzed to assess individual contributions of parameters (temperature or oxidative stress – level and duration) to the bacteria (together and individually). Graphs depicting relative fold expression for each treatment were derived using the ΔΔC_T_ formula, with normalization against the 16S rRNA gene expression at 30°C.

## Results

### Gene Expression of Virulence-Associated Genes in *L. borgpetersenii* Jules and *L. interrogans* Portlandvere Exposed to Temperature Stress Conditions

For *L. borgpetersenii* serovar Jules, 12 (85.7%) of the 14 virulence associated genes were detected at 30°C and included adhesins *lenA*, *lsa21* and *loa22*; haemolysins *sph2* and *tlyC*; OMP porin *ompL1*; invasin *invA*; OMP lipoproteins *lipL32*, *lipL36*, *lipL41*, and *lipL45* and the *fliY* gene involved in chemotaxis. For *L. interrogans* serovar Portlandvere, 13 (92.9%) of the 14 virulence associated genes were also amplified and included all except *ligB*.

**FIGURE 1:**
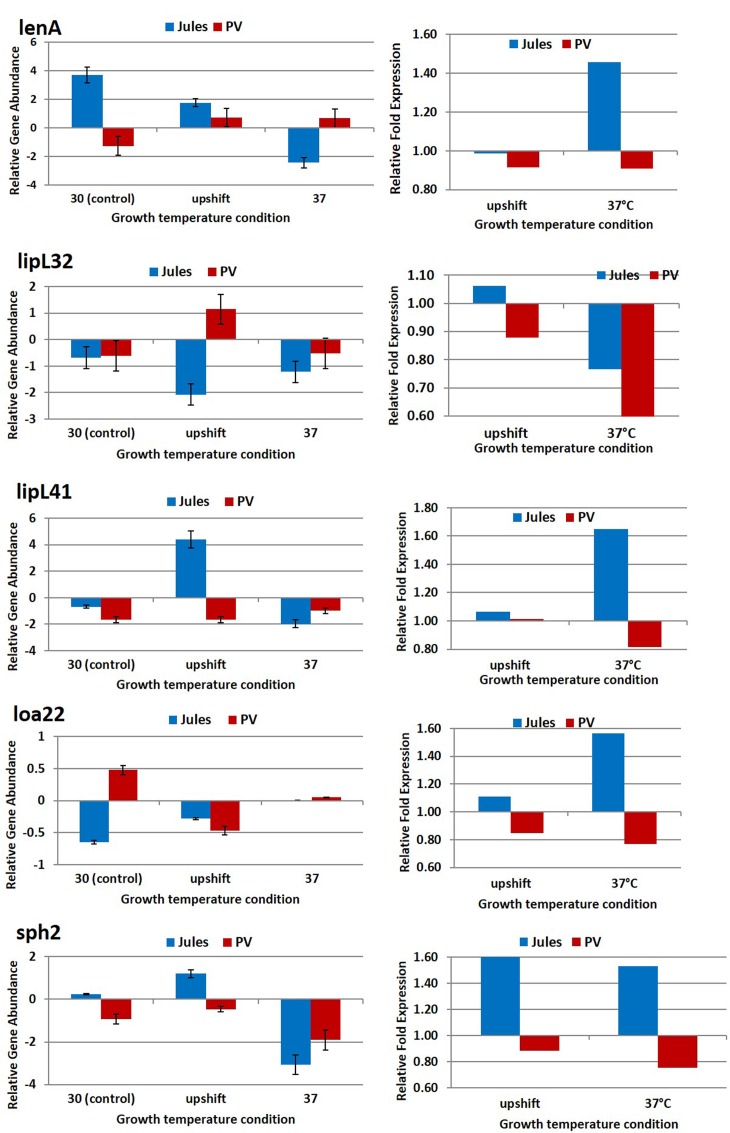
**Comparative analysis of the effects of temperature on transcription of five virulence associated genes in *Leptospira* Jules and *Leptospira* Portlandvere (PV) using qRT-PCR.** Each gene is represented as relative gene abundance (based on log10-transformed gene expression data) with error bars (standard error of mean) for bacteria exposed to 30°C, upshift conditions, and 37°C. Gene expression of 16S rRNA at 30°C served as controls for the purpose of normalization of gene expression at upshift and at 37°C conditions, and calculation of fold changes (shown on the right-hand side).

The expression of five genes, *lenA, lipL32*, *lipL41, loa22*, and *sph2* in Jules and Portlandvere were selected for quantification determination by quantitative real time RT-PCR. As illustrated in **Figure 1** and detailed in **Table 2**, in most cases, temperature stress resulted in significant changes in gene expression within species or between species (Jules vs. Portlandvere). Firstly, we observed varying degrees of decreased expression of *lipL32, loa22*, and *sph2* in Jules at 30°C compared to 37°C, while the expression of *lipL41* was unchanged with the elevation of temperature. In Portlandvere, the increased expression of *lenA* at 37°C compared to 30°C, contrasts that of *loa22* and *sph2* where decreased gene expression was observed. Secondly, in Jules exposed to upshifted temperature conditions compared to 30°C, expression levels of *lipL41, loa22*, and *sph2* were similarly higher, while those for *lenA* and *lipL32* decreased in bacteria at 30°C compared to upshifted conditions. Thirdly, expression levels of all genes were increased, except for *loa22* in Portlandvere at 30°C compared to upshifted conditions. Fourthly, only *lipL32* (in Jules) and *lipL41* (in Portlandvere) expression showed increased expression at 37°C compared to upshifted conditions. Overall, expression of the virulence-associated genes observed in Portlandvere was generally lower relative to expression in Jules. When inter-species comparisons were carried out, we noted that at 30°C, there were higher levels of expression of *lenA* and *sph2* (in Jules compared to Portlandvere) and *loa22* (in Portlandvere compared to Jules). However, at upshifted temperatures, *lenA, sph2*, and *lipL41* were expressed at a higher level in Jules compared to *lipL32* in Portlandvere. Finally, expression of *lenA* was significantly elevated in Portlandvere at 37°C compared to Jules.

**Table 2:** *P*-values associated with comparative analysis of the effects of temperature on transcription of five virulence associated genes in *L*. Jules and *L*. Portlandvere using qPCR.

Strain	Jules			Portlandvere			Jules vs. Portlandvere					
			
	30°C vs. 37°C	30°C vs. upshift	upshift vs 37°C	30°C vs. 37°C	30°C vs. upshift	upshift vs. 37°C	30°C	upshift	37°C	30°C vs. 37°C	30°C vs. upshift	upshift vs. 37°C
*lenA*	**0.0002**	**0.004**	**0.0002**	**0.0008**	**0.0002**	0.294	**0.0048**	**0.001**	**0.0009**	0.212	0.587	0.191
*sph2*	**0.0002**	**0.001**	**0.0001**	**0.005**	**0.004**	**0.0019**	**0.0016**	**0.0002**	0.3323	0.629	0.718	0.420
*lipL41*	**0.001**	**<0.0001**	**<0.0001**	**0.018**	0.464	**0.02**	0.052	**0.01**	0.0736	0.709	0.434	0.405
*lipL32*	**0.0089**	**0.0004**	**0.0044**	0.14	**0.0009**	**0.0017**	0.095	**0.016**	**0.0125**	0.642	0.908	0.974
*loa22*	**0.0028**	0.76	0.1	**0.0021**	**0.0279**	0.0948	**0.006**	0.560	**0.0459**	0.269	0.841	0.318


*Upshift, growth at 30°C followed by growth at 37°C. Significant *p* values are highlighted in bold.*

The greatest fold increases in gene expression was observed for *lenA, lipL41*, and *loa22* (1.4–1.6-fold) in Jules at 37°C and *sph2* (1.5–1.6-fold) in Jules at upshift and 37°C.

### Qualitative Gene Expression of Virulence-Associated Genes in *L. borgpetersenii* Jules and *L. interrogans* Portlandvere Exposed to Oxidative Stress Conditions

Hydrogen peroxide, H_2_O_2_, a ROS, causes alterations in cellular redox potential, where small perturbations stimulate the cell’s anti-oxidant system and larger changes result in apoptosis and necrosis. H_2_O_2_-induced cytotoxicity derived from destabilization of cellular components such as DNA, proteins, and lipids, is enhanced by its lipid solubility which facilitates diffusion across cellular membranes. However, much of the damage caused by ROS occurs within the DNA structure to effect base damage and DNA nicking, leading to mutations.

Generally, growth at 37°C coupled with hydrogen peroxide-induced oxidative stress resulted in gene expression significantly higher in *L. interrogans* Portlandvere compared to *L. borgpetersenii* Jules. This increase in gene expression was more noticeable in upshifted Portlandvere compared to bacteria at 30 and 37°C.

Specifically, differential gene expression under oxidative conditions yielded expression of *loa22, lipL32*, and *lipL41* in both Jules and Portlandvere with *fliY and lsa21* transcripts observed solely in Portlandvere. Collective gene expression among upshifted Jules and Portlandvere yielded more transcripts compared to growth at 30 and 37°C, with growth at 37°C yielding the least gene transcripts. This was particularly noticeable under conditions of low oxidative intensity, 1 mM H_2_O_2_ and varied durations of 30 and 60 min oxidative exposure. Of note, *lsa21*, transcribed in Portlandvere, appeared to be preferentially expressed at low oxidative intensity, regardless of oxidative duration in bacteria grown at 30°C and upshifted temperature conditions. Thus, it is possible that *lsa21* transcription may be both temperature-sensitive (negligible detection at long term growth at 37°C) and oxidative stress-sensitive (detection at ≤1 mM H_2_O_2_). Similar to *lsa21, fliY* expression may be temperature sensitive as long term growth at 37°C yielded reduced *fliY* expression in Portlandvere compared to transcription at all oxidative conditions in bacteria grown at 30°C and upshifted temperatures. Further, neither gene was visibly expressed at 30°C in Portlandevere, suggestive of reduced responsiveness to oxidative stress.

Oxidative stress conditions resulted in differential gene expression: in Portlandvere, *loa22* and *lipL32* were expressed in all 12 combinations of growth temperature, oxidative intensity and duration compared to Jules, however, at 30°C *loa22* was preferentially expressed at low oxidative intensity regardless of duration. On the other hand, the expression of *loa22* was sensitive to oxidative duration at upshifted temperature conditions in Jules, as it was expressed at 60 min duration regardless of intensity.

### Quantitative Gene Expression of Virulence-Associated Genes in *L. borgpetersenii* Jules and *L. interrogans* Portlandvere Exposed to Oxidative Stress Conditions

The quantitative effect of oxidative stress on the expression of the five selected virulence-associated genes were analyzed by trend analysis for bacteria exposed to three temperature conditions, 30, 37, and upshifted conditions, each with subsequent independent exposure to the four hydrogen peroxide-induced oxidative stress states of 1 mM peroxide for 30 min (low oxidative intensity/short durational exposure); 10 mM peroxide for 30 min (high oxidative intensity/short durational exposure); 1 mM peroxide for 60 min (low oxidative intensity/long durational exposure) and 10 mM peroxide for 60 min (high oxidative intensity/long durational exposure. Gene expression in unexposed cultures at the respective growth temperatures were used as comparative baseline control values.

**FIGURE 2:**
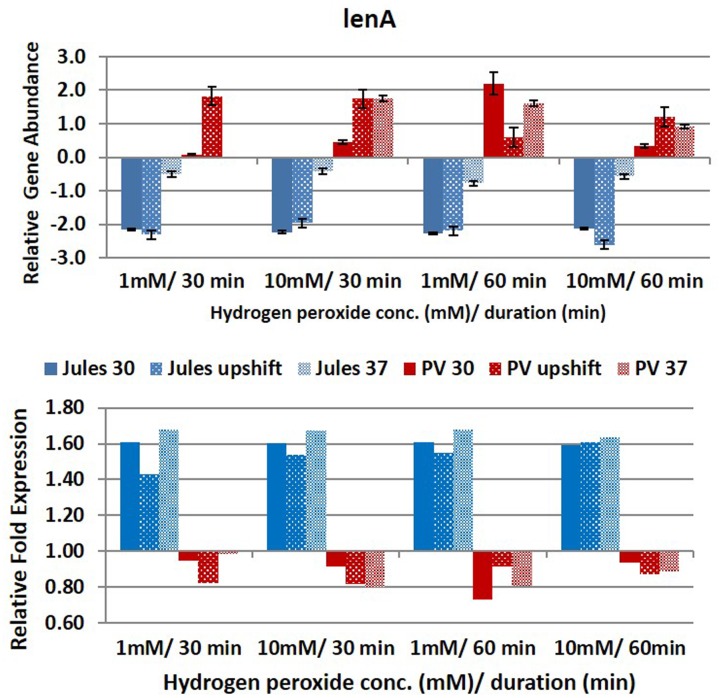
**Comparative analysis of the effects of peroxide-induced oxidative stress on transcription of gene *lenA* in *L*. Jules and *L*. Portlandvere (PV) using qRT-PCR.** The gene is represented as relative gene abundance (based on log10-transformed gene expression data) with error bars (standard error of mean) for bacteria exposed to hydrogen peroxide at 1 mM/30 min, 10 mM/30 min, 1 mM/60 min, 10 mM/60 min, at the three temperature conditions. Gene expression of 16S rRNA at 30°C (without oxidative stress) served as controls for the purpose of normalization of gene expression in the presence of oxidative stress at the various combinations of exposures, and calculation of fold changes (shown below).

**FIGURE 3:**
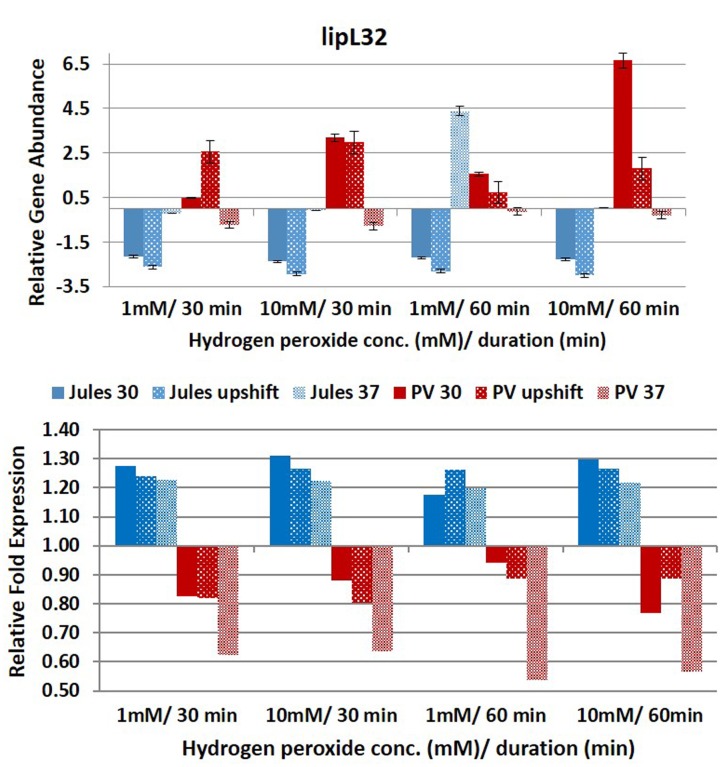
**Comparative analysis of the effects of peroxide-induced oxidative stress on transcription of gene *lipL32* in *L*. Jules and *L*. Portlandvere (PV) using qRT-PCR.** The gene is represented as relative gene abundance (based on log10-transformed gene expression data) with error bars (standard error of mean) for bacteria exposed to hydrogen peroxide at 1 mM/30 min, 10 mM/30 min, 1 mM/60 min, 10 mM/60 min, at the three temperature conditions. Gene expression of 16S rRNA at 30°C (without oxidative stress) served as controls for the purpose of normalization of gene expression in the presence of oxidative stress at the various combinations of exposures, and calculation of fold changes (shown below).

**FIGURE 4:**
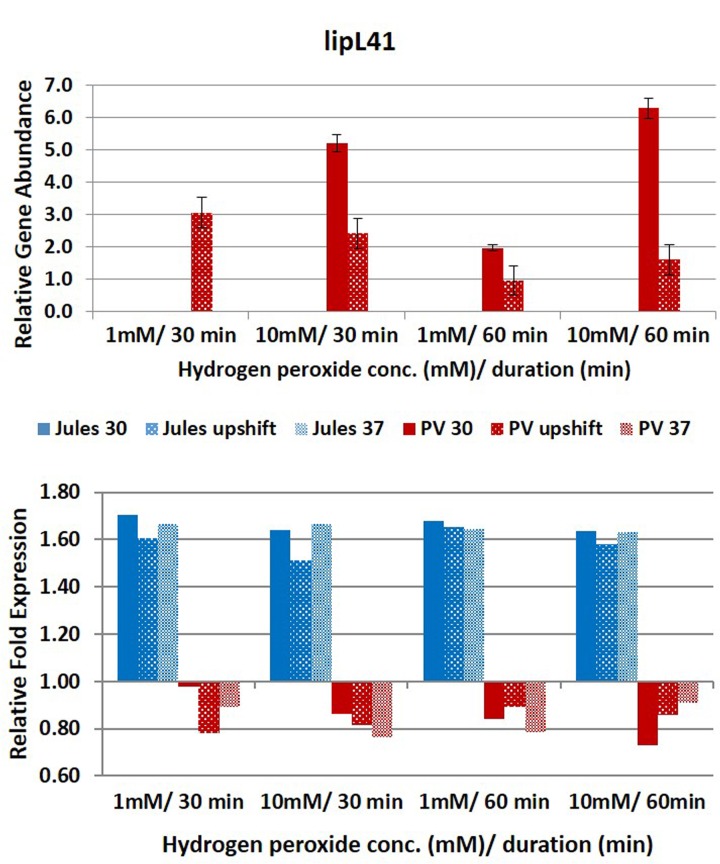
**Comparative analysis of the effects of peroxide-induced oxidative stress on transcription of gene *lipL41* in *L*. Jules and *L*. Portlandvere (PV) using qRT-PCR.** The gene is represented as relative gene abundance (based on log10-transformed gene expression data) with error bars (standard error of mean) for bacteria exposed to hydrogen peroxide at 1 mM/30 min, 10 mM/30 min, 1 mM/60 min, 10 mM/60 min, at the three temperature conditions. Gene expression of 16S rRNA at 30°C (without oxidative stress) served as controls for the purpose of normalization of gene expression in the presence of oxidative stress at the various combinations of exposures, and calculation of fold changes (shown below).

**FIGURE 5:**
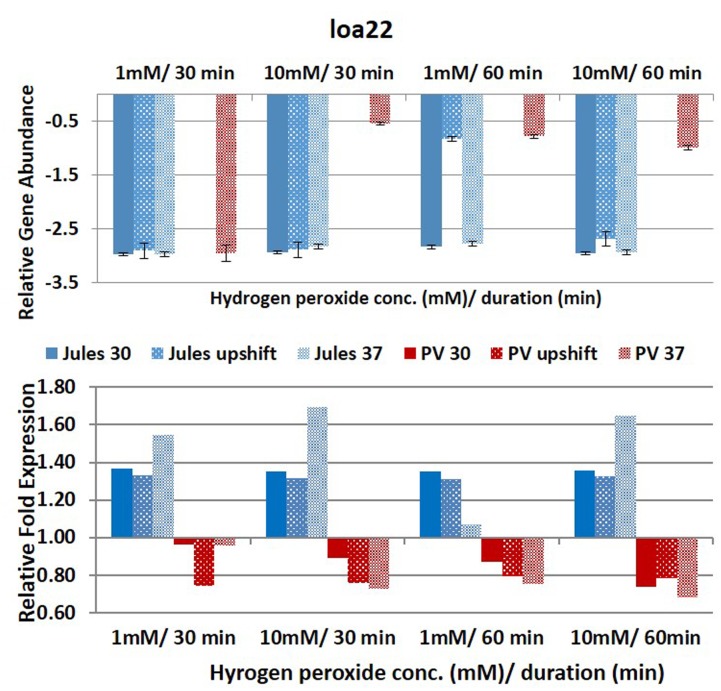
**Comparative analysis of the effects of peroxide-induced oxidative stress on transcription of gene *loa22* in *L*. Jules and *L*. Portlandvere (PV) using qRT-PCR.** The gene is represented as relative gene abundance (based on log10-transformed gene expression data) with error bars (standard error of mean) for bacteria exposed to hydrogen peroxide at 1 mM/30 min, 10 mM/30 min, 1 mM/60 min, 10 mM/60 min, at the three temperature conditions. Gene expression of 16S rRNA at 30°C (without oxidative stress) served as controls for the purpose of normalization of gene expression in the presence of oxidative stress at the various combinations of exposures, and calculation of fold changes (shown below).

**FIGURE 6:**
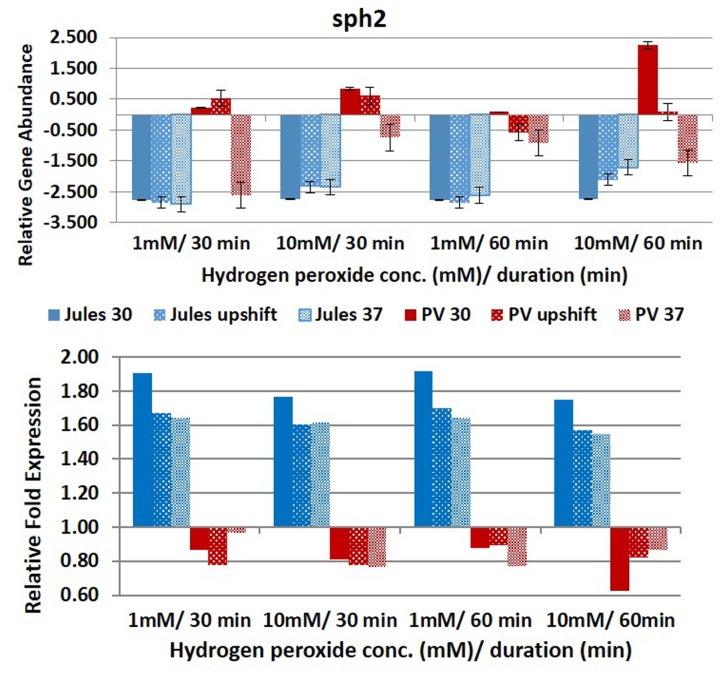
**Comparative analysis of the effects of peroxide-induced oxidative stress on transcription of gene *sph2* in *L*. Jules and *L*. Portlandvere (PV) using qRT-PCR.** The gene is represented as relative gene abundance (based on log10-transformed gene expression data) with error bars (standard error of mean) for bacteria exposed to hydrogen peroxide at 1 mM/30 min, 10 mM/30 min, 1 mM/60 min, 10 mM/60 min, at the three temperature conditions. Gene expression of 16S rRNA at 30°C (without oxidative stress) served as controls for the purpose of normalization of gene expression in the presence of oxidative stress at the various combinations of exposures, and calculation of fold changes (shown below).

Selected genes were differentially expressed by Jules and Portlandvere as temperature changes had a measureable impact on the transcriptional response observed for *lipL41, lipL32*, and *loa22* (**Figures 2**–**6**). For each gene analyzed, the fold change in expression was illustrated below the relative gene abundance plots. Specifically, *lenA* expression was significantly reduced in Jules at low oxidative intensity (*p* = 0.007) and between exposure times (*p* = 0.02). The other gene that had significant change was *sph2* in Jules for short (*p* = 0.004) and long exposure (*p* = 0.02), and between low and high intensities (*p* = 0.003). When expression in Jules was compared with that in Portlandvere, we noted that all genes except *lipL41* were significantly different.

With the complexity of the oxidative stress response so intricately linked with heat stress response, it was not surprising to note that oxidative intensity and duration were influential at upshifted and elevated growth temperatures. The intensity of H_2_O_2_-induced oxidative stress was an essential element in both *lenA* and *sph2* expressions. An increase in oxidative stress intensity from 1 to 10 mM H_2_O_2_ in Jules at 37°C and upshifted conditions yielded increased *sph2* and *lenA* expression, respectively. Further, increased duration of exposure from 30 to 60 min resulted in decreased *lenA* transcription among upshifted cultures of Jules.

Compared to Jules where temperature was instrumental in only two of five genes, temperature was an essential element in four of five genes (*lipL32, lipL41, loa22*, and *sph2*) in Portlandvere subjected to oxidative stress. A temperature change from 30°C to 37°C was important for the decreased expressions of *lipL32* (*p* = 0.0280) and *sph2* (*p* = 0.0342) but extremely important in the reduced transcription of *loa22* (*p* < 0.0001), much lower in Portlandvere at 37°C and upshifted temperatures. *Sph2* transcripts were significantly lower in Portlandvere at 30°C (*p* = 0.0342), particularly following 60 rather than 30 min of oxidative exposure and 10 mM H_2_O_2_ rather than low oxidative intensity. Temperature changes from 30°C to upshift were more important in decreased *loa22* expression (*p* = 0.0007) whereas changes from upshift to 37°C were impactful on decreased *lipL41* (*p* = 0.0119) and *lipL32* (*p* = 0.0304) transcripts in Portlandvere. No other combinations, when analyzed, were significantly different.

At low oxidative intensity, the interplay between genetic predisposition and temperature was biased towards Portlandvere particularly when grown at 30°C and upshifted temperatures (**Figures 2–6**). Under these conditions, higher *lenA*, *lipL32*, *lipL41*, and *sph2* transcripts were observed in Portlandvere compared to Jules grown at the corresponding temperatures. Further, increased oxidative intensity (10 mM peroxide) was considered an essential element in intra-comparative differential expressions, as all five genes were expressed several fold higher in Portlandvere when compared to the respective cultures of Jules. This was particularly evident at high oxidative intensity/long duration for *lipL32*, *lipL41*, *loa22*, and *sph2* gene expression.

In all cases, fold increases in expression of genes was positive (20–70%) for Jules subsequent to normalization with expression data for the 16S rRNA gene.

## Discussion

The transmission cycle of pathogenic *Leptospira* species necessitates the ability to respond to environmental changes and is supported by altered gene expression of leptospiral exoproteins predominantly involved in motility, signal transduction and energy-generating functions during infection (Matsui et al., 2012; Eshghi et al., 2015). This study examined the effects of temperature and oxidative stress on virulence associated genes in *L. interrogans* serovar Portlandvere and *L. borgpetersenii* serovar Jules. Several studies have demonstrated differential gene transcription in leptospires transitioned from environmental to host simulated conditions involving changes in temperature (Lo et al., 2006, 2009), osmolarity (Matsunaga et al., 2007); serum exposure (Patarakul et al., 2010), and macrophage interactions (Xue et al., 2010).

In this study, the variability in gene expression may possibly be attributed to any individual and/or combination of growth temperature, strain diversity, oxidative conditions or other unknown factors. In most cases, temperature by itself resulted in significant changes in gene expression within the individual strains and between the strains for the five genes analyzed in this study. Not surprisingly, but noteworthy, there was generally higher transcription in Portlandvere compared to Jules (notwithstanding the higher fold in Jules increases relative to 16S rRNA expression). This supports reports of loss of gene function involved in environmental sensing and metabolic transport and utilization in *L. borgpetersenii* (Picardeau, 2015), possibly due to the presence of more pseudogenes (around 12%) in *L. borgpetersenii* compared to <4% in *L. interrogans*, and gene reduction in *L. borgpetersenii* (Bulach et al., 2006). The approximately 700 kb reduced genome of *L. borgpetersenii*, being about 16% smaller than *L. interrogans*, exemplifies a restrictive lifecycle of a host to host mode of transmission compared to the adaptability to either aqueous or mammalian host environs for the larger sized genome of *L. interrogans*. The lack of expression of *ligB* or *mce* using both genomic and expression analysis may be due in part to loss or attenuation of these genes in highly passaged cultures, lack of ORFs and/or regulatory dysfunction. In virulent leptospiral strains, *ligB* has been shown to be upregulated upon exposure to temperature and osmolarity, with its expression lost in high passaged cultures (Bulach et al., 2006). The eleven and nine genes that were negligibly detected using endpoint RT-PCR in Jules and Portlandvere, respectively, may have possibly been expressed below a detectable threshold; subject to amplification failure, pre- or post-transcriptional modifications in the highly passaged cultures and/or responsive to factors other than temperature, oxidative intensity and duration. Differential gene expression in Jules was observed across conditions of low and high oxidative stress intensities for both short and long durations of oxidative stress. We propose that under oxidative stress, the expressions of *loa22, lipL32*, and *lipL41* in Jules are co-regulated by temperature and oxidative stress, as their expressions were reduced at 37°C, and oxidative conditions of long duration yielded the most abundant transcription when the bacteria were grown at 30°C and upshifted temperatures. For Portlandvere, temperature played a greater role in differential gene expression compared to oxidative stress and duration.

Notwithstanding lower *lenA* expression in Jules, it was clear that there was co-regulation by temperature and oxidative stress in Portlandvere, evidenced by better yields at upshifted temperature vs 30°C, at 10 mM (high oxidative intensity) vs. 1 mM H_2_O_2_ (low oxidative intensity), and at 30 min vs 60 min. Interestingly, in the absence of oxidative stress, differential *lenA* expression in Portlandvere was particularly enhanced at upshifted temperatures and at 37°C compared to 30°C and increased following 30 min oxidative duration, particularly at high oxidation intensity. While the specific function of *lenA* remains unclear, it is reported to facilitate adherence to host ECM and plasma components to result in degradation of fibrinogen, connective tissue and immunoglobulin (Verma et al., 2010), suggestive of a putative role in leptospiral dissemination and/or evasive strategies. The finding of higher *lenA* transcripts among upshifted cultures of Portlandvere alludes to the possible involvement at early onset of infection possibly in host recognition to facilitate adhesion, bind plasminogen, overcome host derived ROS, and/or other unknown functions.

In the present study, the expression of the calcium-mediated LipL32, one of several plasminogen binding leptospiral OMPs (Picardeau, 2015), was influenced by temperature and the duration of oxidative stress. Temperature was an essential factor in *lipL32* expression in both Jules and Portlandvere where changes from 30°C to upshift conditions resulted in increased and decreased *lipL32* transcription in Portlandvere and Jules, respectively. Synergistic changes in temperature and the duration of oxidative stress may also possibly co-regulate *lipL32* in Portlandvere, as long rather than short duration was important in the increased *lipL32* expression in Portlandvere at 30°C and upshift conditions. This suggests that *lipL32* is responsive to oxidative stress for Portlandvere, which contrasts down-regulation of LipL32 upon macrophage interaction with *L. interrogans* serovar Lai (Xue et al., 2010) and in in vivo studies using animal models of infection (Matsui et al., 2012). With some 38,000 copies per cell, the immunodominant subsurface lipoprotein LipL32 (Pinne et al., 2012), conserved among pathogenic *Leptospira*, is both highly antigenic and immunogenic, and has been shown to induce a robust inflammatory response via the NF-κB signaling pathway in cultured human and murine renal cells within 2 h via TLR2 activation (Yang et al., 2006; Fitzgerald et al., 2007). This is suggestive of an early inflammatory response likely leading to detrimental effects observed in tubule-interstitial nephritis observed during leptospirosis.

This observation contrasted with decreased *lipL32* expression in Jules at upshift conditions and 37°C, although of lower abundance when compared to Portlandvere and warrants further investigation of possible strain specificity and a putative role for LipL32 in the oxidative stress mediated responses of *L. borgpetersenii* in mammalian hosts. Decreased *lipL32* expression in Jules at 37°C and upshift compared to 30°C, decreased *lenA* and *sph2* among Jules grown at 37°C and upshifted temperatures suggest no putative role during early infection. Because *L. borgpetersenii* is usually transmitted host-to-host transmission where the normal body temperature is ∼37°C, it is likely that the genes are expressed constitutively in Jules with a non-significant regulation by oxidative stress. In light of this, we postulate that lenA might be involved in an anticipatory adaptive response to ‘low dosage pre-exposure’ which facilitate resistance to the damaging effects of host derived H_2_O_2_ (Cabiscol et al., 2000). With more genes involved in signal transduction, regulatory and metabolic processes in the larger sized genome of *L. interrogans*, the role of *lipL32* may be relegated to known functions such as outer membrane stabilization, and adhesion of host cells rather than in oxidative stress response.

Further, the results suggest that possible interplay between oxidative stress duration, temperature and strain diversity may have roles in *lipL41* expression, as the gene was expressed following both short and long durations of oxidative stress of varying oxidative intensity in Portlandvere at 30°C and preferentially following high oxidative intensity. Conversely, differentially expressed *lipL41* displayed temperature sensitivity in Jules, temperature changes from 30°C to upshift were important to yield increased *lipL41* transcripts. However, neither changes in oxidative stress intensity nor duration significantly influenced *lipL41* transcription in Jules. This decline in *lipL41* in Jules as temperature increased from upshift to 37°C, while concurring with similar reports of downregulation of LipL41 upon interaction with macrophage derived cells of *L. interrogans* at 37°C (Xue et al., 2010), does contrast with other reports which chronicle expression of LipL41 in infection and the urine of rats. On the other hand, the minimal *lipL41* expression at upshifted temperatures and the undetectable *lipL41* transcripts among *L. borgpetersenii* Jules grown at 30°C and 37°C concurred with findings of Lo et al. (2009), indicating unaltered *lipL41* expression at 30°C and 37°C. This may be suggestive of regulatory factors other than oxidative stress as indicated by Cullen et al. (2004) and Matsui et al. (2012), or lack of co-transcription of the *lep* chaperone (King et al., 2013).

Expression of *loa22*, the second most abundant leptospiral OMP (Ristow et al., 2007; Zhang et al., 2010), was noteworthy in Portlandvere compared to Jules, particularly when grown at 30°C and even more so following high oxidative intensity. Temperature was an essential factor in *loa22* expression in Portlandvere, where changes from 30°C through to upshift to 37°C resulted in decreased *loa22* transcription in Portlandvere. *Loa22* sensitivity to intensity of oxidative stress may allude to possible co-regulation as higher yields were observed following treatment with 10mM H_2_O_2_ in Portlandvere. One study reported modest downregulation upon macrophage interaction with serovar Lai (Xue et al., 2010) while another attributed differences between leptospiral strains and macrophages leading to strain-specific interactions (Toma et al., 2011). Similarities between leptospiral OmpA-like, Loa22 and OmpA in *E. coli* suggest that Loa22 may be osmoregulated, growth rate/phase dependent, and with reduced expression at lower than optimal temperatures (Smith et al., 2007).

Increased *lipL32* and *loa22* transcription in Portlandvere under conditions of oxidative stress observed during this study were converse to down-regulation in *L. interrogans* Lai reported by Xue et al. (2010). In fact, in that study, alterations in the outer membrane of Lai upon interaction with macrophages resulted in a highly downregulated clade 1 consisting of major OMPs, *lipL41, ompL1, lipL32, lipL48*, and *ompL47* and a moderately downregulated clade 2 comprising *lipL45* and *loa22*, among other genes.

Differential *sph2* transcription was observed in Jules vs Portlandvere with possible co-regulation by temperature in Jules (growth at upshift gave better yields vs growth at 30°C or 37°C), or temperature and oxidative intensity in Portlandvere (high oxidative intensity gave better yields than low intensity). For this gene, the duration of oxidative stress was not considered a significant regulatory factor for Jules. Further, temperature by itself was not an important driver and contrasted with the findings of other thermo-regulatory studies by Qin et al. (2006) and Eshghi et al. (2015) where lowered *sph2* expression was observed at 37°C. Other studies have shown upregulation of magnesium-sensitive *sph2* in the presence of physiological osmolarity (Matsunaga et al., 2007) and during infection (Narayanavari et al., 2015). SphH in *L. borgpetersenii* and Sph2 in *L. interrogans* share >50% structural similarity to Smase C of pathogenic *Listeria ivanovii* and the beta toxin of *Staphylococcus aureus* (Narayanavari et al., 2012), which mediate escape from phagocytic vacuoles to release the bacterial cells in the cytosol (Gonzálvez-Zorn et al., 1999). Notwithstanding the significant differential expressions observed in this study, we cannot rule out the possibility of observations due to the differences in experimental temperature, harvesting time, source and complexity of oxidative stress (i.e., simplified H_2_O_2_ induction versus macrophage interaction).

## Conclusion

Differential gene expressions corresponding with temperature changes from 30°C to upshift; 30°C to 37°C and upshift to 37°C and responsiveness to increased intensity and duration of oxidative stress were of keen interest and prompt further investigation of possible role during infection. With an appreciation of the complexity of integrated mechanisms, and genes and gene products involved in the oxidative response of a cell, the in vitro conditions of this study were not meant to simulate physiological conditions of the complex system of oxidative stress, however, the results serve as an important snapshot of selected gene expression in response to temperature and oxidative stress. While it is clear that expression of many virulence genes in highly passaged strains of *Leptospira* are attenuated or lost, genetic predisposition, changes in growth temperature and/or oxidative intensity and/or duration were factors which acted in isolation or together with other regulatory cues to contribute to the variable gene expression observed in this study. Overall, differential gene expression in serovar Portlandvere was more responsive to temperature and oxidative stress, although relative to 16S rRNA, fold increases in gene expression were associated with Jules.

## Author Contributions

TF assisted in the design of the study, carried out the expression assays, performed statistical analyses and drafted the manuscript. PB conceived of the study, its design and coordination and edited the manuscript. Both authors read and approved the final manuscript.

## Conflict of Interest Statement

The authors declare that the research was conducted in the absence of any commercial or financial relationships that could be construed as a potential conflict of interest.
